# Reinforcement learning driven edge–cloud coordination for secure and energy efficient IoMT

**DOI:** 10.3389/fdgth.2026.1824480

**Published:** 2026-06-19

**Authors:** Santhos Kumar Sasikumar, Tarun Vinod Pai, Kumaran Kalidasan, Saranya Gajendran

**Affiliations:** Vellore Institute of Technology, School of Computer Science and Engineering, Chennai, Tamil Nadu, India

**Keywords:** reinforcement learning, edge computing, cloud computing, Proximal Policy Optimization (PPO), energy optimization

## Abstract

The Internet of Medical Things (IoMT) enables sophisticated medical devices, but it also poses significant challenges in terms of data privacy, real-time processing, and energy efficiency for edge devices with limited resources. In this paper, we propose a hierarchical framework for intelligent and secure IoMT-based healthcare monitoring. At the sensor nodes, Federated Variational Mode Decomposition (VMD) is used to decompose physiological signals and locally extract high-fidelity features, ensuring data privacy. To overcome the computational limitations of microcontroller- based sensor nodes, a SparseBonsai neural network is designed for real-time classification of medical signals on the sensor nodes. A centralized orchestration layer, controlled by a Proximal Policy Optimization (PPO) reinforcement learning agent, makes dynamic decisions on whether to queue data for low-latency processing at the edge server or offload to the cloud, depending on data severity, network conditions, and battery level. To further improve energy efficiency, an advanced Sha-Dragon (Shannon-Entropy Dragonfly) optimization algorithm is proposed for resource and transmission power allocation in the IoT network. For security, a dual-layer approach is adopted: ASCON v1.2 lightweight authenticated encryption is used to secure node-to-edge communications, and a WireGuard VPN with ChaCha20-Poly1305 encryption protects data in transit to the cloud. Experimental validation on a Raspberry Pi 5 testbed with a cloud-connected laptop shows that the proposed system achieves a significant reduction in latency for critical alerts and improves the battery life of IoT nodes (8.5 days) compared to the conventional non-adaptive offloading approach. The results confirm the effectiveness of the proposed framework to facilitate energy-efficient, privacy-preserving, and real-time healthcare monitoring in IoMT.

## 1 Introduction

With the emergence of Fifth Generation (5G) telecommunications and the rapid miniaturization of sensing technologies, a significant paradigm shift is occurring in the healthcare industry through the Internet of Medical Things (IoMT). The IoMT paradigm enables interconnected medical devices and continuous health monitoring systems, transforming traditional reactive healthcare into proactive and real-time patient-centric services, as discussed by Sadiku et al. and Khan et al. Market forecasts further indicate that the number of connected IoT devices will exceed 50 billion in the near future, leading to an unprecedented growth in physiological data such as electrocardiograms (ECG) and wearable sensor streams, as reported by Ericsson. In this evolving “Smart Healthcare 4.0” ecosystem, applications such as real-time arrhythmia detection, automated drug delivery, and remote robotic surgery demand ultra-low latency, high reliability, and strict data privacy compliance, as highlighted by Coburn et al. However, the inherent resource constraints of IoMT devices, including limited battery capacity and computational capability, make it impractical to execute complex deep learning algorithms directly on sensor nodes.

To address these limitations, the computing paradigm has shifted towards a multi-tier architecture integrating Mobile Edge Computing (MEC) with cloud infrastructures. MEC enables computation offloading from resource-constrained devices to nearby edge servers, thereby reducing latency and improving responsiveness, as surveyed by Mao et al. and Wang et al. Despite these advancements, task offloading in IoMT environments remains a complex problem due to the stochastic nature of task arrivals and the trade-off between latency and energy consumption. Traditional approaches rely on static heuristics or centralized brokers, which are insufficient for handling highly dynamic network conditions. Consequently, recent research has explored Deep Reinforcement Learning (DRL) techniques to enable adaptive decision-making, with foundational work by Mnih et al. demonstrating the effectiveness of Deep Q-Networks (DQN) for dynamic control tasks. However, value-based methods such as DQN and its variants suffer from instability and overestimation bias, while improved approaches like Twin Delayed Deep Deterministic Policy Gradient (TD3), proposed by Fujimoto et al., still face challenges in convergence stability under highly dynamic and sensitive healthcare scenarios.

Another critical limitation in existing IoMT offloading frameworks is the lack of robust privacy- preserving mechanisms. Most conventional models follow a “transmit-then-process” paradigm, where raw patient data is transmitted to centralized edge or cloud servers, thereby exposing sensitive medical information to potential security breaches. As highlighted by Mahmood et al., such centralized architectures introduce single points of failure and significant privacy risks. This necessitates the development of decentralized, privacy-aware frameworks that ensure data confidentiality at the source while maintaining system performance.

To overcome these challenges, this paper proposes a hierarchical and privacy-preserving IoMT framework that integrates signal processing, lightweight learning, reinforcement learning, and secure communication. Specifically, a Federated Variational Mode Decomposition (VMD) approach is employed at the sensor level to extract intrinsic mode functions and generate feature representations locally, thereby avoiding transmission of raw physiological data, consistent with prior VMD-based signal processing approaches by Dragomiretskiy and Zosso and further advancements summarized by Wang et al. These extracted features are then classified using a lightweight SparseBonsai neural network, enabling real-time inference on resource-constrained devices. The physiological data used in this study is based on standard ECG datasets such as the MIT-BIH Arrhythmia Database (1), widely used for heartbeat classification tasks as demonstrated by Moody and Mark and further explored by Zhang et al.

At the architecture layer, a Proximal Policy Optimization (PPO) agent is employed to dynamically manage task offloading decisions. Unlike value-based DRL methods, PPO, introduced by Schulman et al., provides stable policy updates through a clipped surrogate objective function, making it well-suited for real-time and safety-critical healthcare environments. The PPO agent evaluates multiple system states, including data severity, network conditions, and device battery levels, to decide whether tasks should be processed locally at the edge for low latency or offloaded to the cloud for long-term storage and analysis. Additionally, to optimize network resource utilization, a novel Sha-Dragon optimization algorithm is introduced, combining Shannon entropy with the Dragonfly algorithm to achieve a balanced exploration–exploitation trade-off for efficient transmission power and bandwidth allocation.

Finally, to ensure end-to-end data security, a dual-layer security mechanism is incorporated. Lightweight ASCON v1.2 authenticated encryption is used for secure communication between sensor nodes and edge devices, while a WireGuard VPN employing ChaCha20-Poly1305 encryption ensures secure data transmission between the edge and cloud layers. This integrated framework addresses key challenges in IoMT systems, including energy efficiency, latency optimization, privacy preservation, and secure communication, thereby enabling a robust and scalable solution for next-generation healthcare monitoring applications.

## 2 Related works

This section reviews the existing literature on task offloading, signal decomposition, and Deep Reinforcement Learning (DRL) in edge computing environments. Specifically, we analyze state-of-the- art methodologies that utilize Variational Mode Decomposition (VMD) and various DRL architectures, highlighting the critical research gaps that motivate the proposed Federated VMD-Attention TD3 (FVAT) framework.

### 2.1 Signal decomposition and task classification

Efficient task scheduling in heterogeneous IoMT environments requires accurate traffic classification to prioritize critical health data. Recent advancements have moved beyond simple heuristic-based classification toward signal processing techniques. Kumaran and Sasikala (2) introduced a Variational Mode Decomposition (VMD) approach optimized by Random Forest (RF) to classify edge application activities. Their method decomposes predictor variables into Band-Limited Intrinsic Mode Functions (BL-IMFs) to isolate time-sensitive and priority-sensitive workloads. This VMD-RF architecture significantly improved scheduling rates by classifying tasks based on parameters like task length, network demand, and delay sensitivity. However, this approach relies on a static classification pipeline where the penalty function and the number of modes K must be predetermined or manually tuned. While effective for classifying discrete task types, this method does not dynamically weight the importance of specific sub-signals in real-time during the decision-making process, a limitation that the proposed Attention-based mechanism aims to address.

### 2.2 Deep reinforcement learning for task offloading

To manage the high-dimensional state spaces of MEC networks, DRL has become the standard for dynamic resource allocation. Two primary streams of DRL algorithms have been dominant: Value-Based Methods (DDQN): Kumaran and Sasikala (2) proposed a Double Deep Q- Network (DDQN) architecture to facilitate offloading decisions. By decoupling the selection and evaluation of actions, DDQN mitigates the overestimation bias inherent in standard DQN. Their implementation demonstrated a 25 percent improvement in average delivery time compared to baseline algorithms like Genetic Algorithms or Particle Swarm Optimization (3).

Actor-Critic Methods (SAC): More recently, Saranya et al. (4) argued that value-based methods are inflexible (4) in real-time scenarios and proposed a Soft Actor-Critic (SAC) network. Their “Novel DRL-TO” strategy utilizes an actor-critic structure where the agent learns optimal allocation by interacting with the environment and storing transitions in a replay buffer. This approach achieved a resource utilization rate of 70 percent and a task completion rate of 93.5 percent.

Despite these successes, both architectures face stability and efficiency challenges. Saranya et al. (4) explicitly noted that Actor-Critic algorithms can be “computationally intensive,” potentially causing delays in real-time decision-making on resource-constrained devices. Furthermore, while DDQN improves stability, it lacks the robustness of policy-gradient methods in continuous action spaces, necessitating the shift toward the Twin Delayed DDPG (TD3) architecture (5) proposed in this study.

### 2.3 Meta-Heuristic optimization in edge computing

Hybrid frameworks that combine DRL with meta-heuristic optimization have shown promise in overcoming local optima. The Dynamic Arithmetic Optimization Algorithm (DAOA) was successfully integrated by Kumaran and Sasikala (2) to select the optimal Computational Access Point (CAP) for offloading. By utilizing dynamic accelerated functions to balance exploration and exploitation, DAOA avoided the need for extensive manual parameter tuning associated with traditional meta-heuristics.

While this demonstrated the efficacy of DAOA for spatial resource selection (i.e., choosing where to offload), its application to the internal hyperparameter tuning of the DRL agent itself remains unexplored in these works. The proposed framework extends DAOA to dynamically optimize the learning rates and aggregation weights of the federated learning process, addressing the training instability observed in static configurations.

### 2.4 Privacy and security gaps in IoMT

A critical deficiency in the current body of work is the handling of sensitive patient data. Saranya and Sasikala (6) highlighted that existing large-scale IoT deployments often overlook security, noting that “the integration of IoT devices in healthcare raises significant concerns” regarding unauthorized access to sensitive patient information. They explicitly stated that current implementations “may not adequately address potential vulnerabilities, such as data breaches”. Similarly, the centralized training approach used in both the DDQN and SAC models requires transmitting state information to a central server, creating a single point of failure.

This research identifies a significant gap: the lack of a Federated Learning approach that can leverage the feature extraction power of VMD and the decision-making stability of DRL without transmitting raw data. The proposed FVAT framework fills this void by keeping raw data local and sharing only model gradients, directly responding to the future work suggested by Saranya et al. (4) regarding the investigation of robust security measures.

## 3 Proposed system

### 3.1 IoMT framework

**Figure 1:**
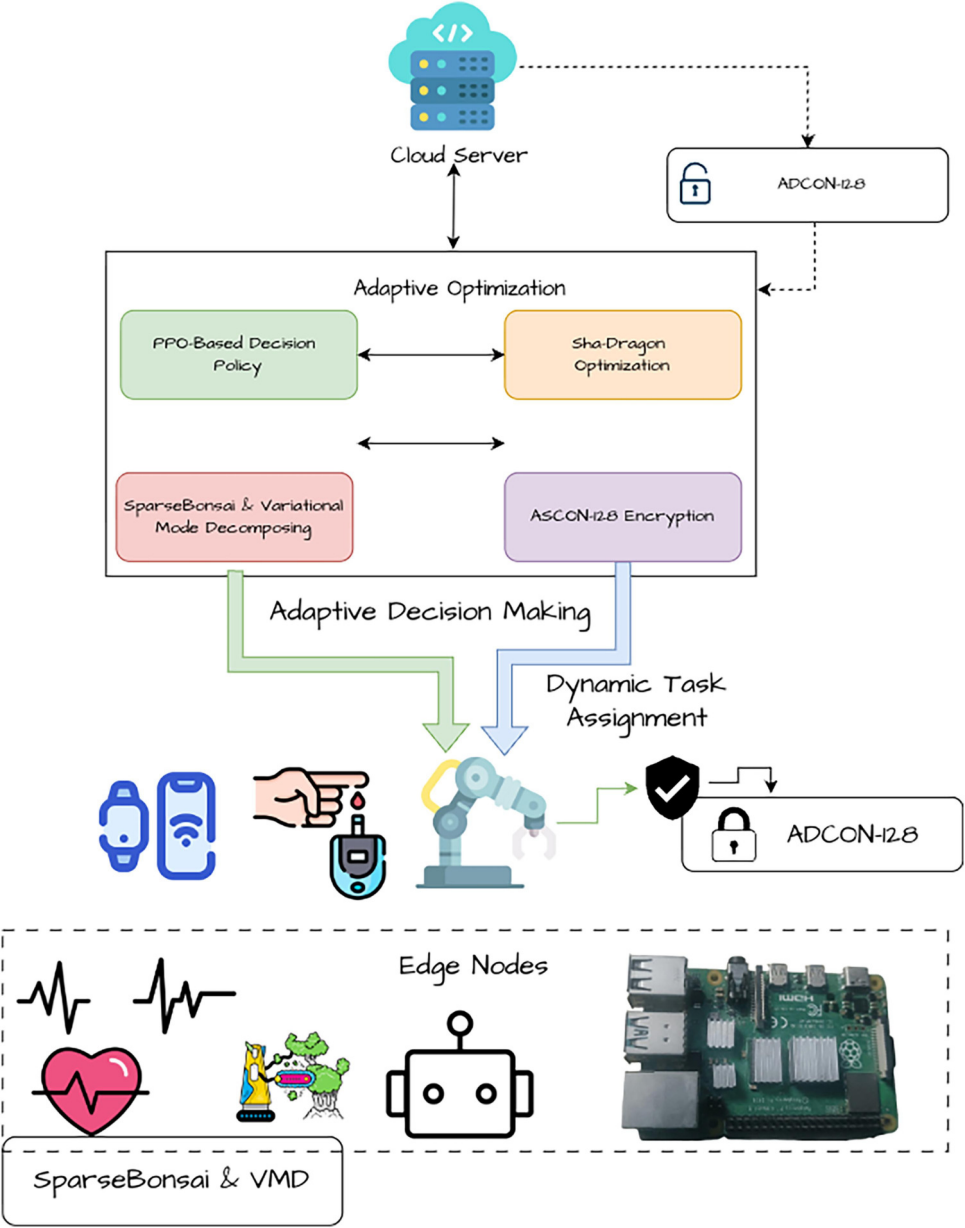
System architecture of the proposed PPO Sha-Dragon enabled edge–cloud MIoT framework. Icons from: “Cloud Server” by juicy_fish, “Robotic Arm” by Freepik, and “Sugar Blood Level” by Freepik, “Shield” by Kiranshastry, “Waveform” by Shashank Singh, “Heart Rate” by Marcus Christensen, “Robot” by Freepik and “Technology” by Roundicons Premium, licensed under Flaticon License. “Smartwatch icon” by Hendi Perkasa (https://www.magnific.com/legal/terms-of-use).

Figure 1 illustrates the proposed three-layer architecture of the Internet of Medical Things (IoMT) that leverages edge intelligence, adaptive task orchestration, and cloud analytics to enable secure and energy- efficient health monitoring. The first layer, named the Medical IoT Layer, consists of diverse wearable and implantable sensing platforms such as ECG electrodes and SpO2 sensors that continuously monitor physiological signals with very low power and privacy budgets. Unlike traditional IoMT architectures that send raw data to remote cloud servers, this design processes critical data locally following a privacy-by- design strategy. (ECG data for our experiments were drawn from the MIT-BIH Arrhythmia Database.)

In this layer, each node of the IoMT network employs Federated Variational Mode Decomposition (Fed-VMD) to locally decompose the raw physiological signals (7) into their intrinsic components and compact feature representations without requiring the transmission of raw physiological data. The features are then evaluated to determine the medical severity level of the observed physiological condition, directly influencing the computation and communication strategies at the next layers. For instance, life-threatening conditions such as arrhythmia detection are labeled as high-severity events (*S* → 1) and require immediate edge inference and notification, while normal physiological patterns are labeled as low-severity events (*S* → 0) and require less urgent processing or deferred analysis. During network outages (e.g., VPN disconnections), edge nodes continue critical processing locally and buffer non-urgent data until connectivity is restored.

### 3.2 Computational task model

Each incoming medical request is modeled as a task with key parameters. Formally, task A is defined as a *A* = (*D*, *C*, *T*_deadline_, *S*) where:

- Data (D): Total bytes required for transmission (e.g., raw ECG waveform)
- CPU Cycles (*C_i_*): The computational complexity required for processing
- Completing deadline (*T_i_*): The maximum allowable latency before the data medically irrelevant, particularly for time-critical emergency tasks
- Severity/Priority (*S_i_*): All the tasks indicating the medical urgency for the computational tasks and results, (0 ≤ *S* ≤ 1), derived from the SparseBonsai classifier (8)

Execution Modes:

- Local Execution: The task is computed entirely on the Raspberry Pi 5. This minimizes communication delays and avoids energy cost for transmission, which is essential for high-S (critical) tasks. This mode is enforced for critical tasks (S ¿ 0.8) to guarantee ultra-low latency and reliability, especially during network disruptions.
- Offload Execution: The task is transferred to the cloud for processing. This is selected when local computational resources are insufficient or when the task is low-priority. By offloading non-urgent workloads to the cloud, the system utilizes abundant remote computing resources while lowering the energy consumption of the Raspberry Pi 5.This mode is typically used for non-urgent workloads (S¡ 0.3) or when local battery reserves are critically low.

By modeling tasks in this structured form, the DRL agent deployed on the Raspberry Pi 5 evaluates D, C, T, and S to dynamically determine the optimal execution mode (local vs. offloading) for each IoMT task under varying system conditions.

### 2 Resource and energy constraints

The edge of our system operates under strict energy–latency trade-offs. Localizing tasks (e.g., executing on all CPU cores at the Pi5 end) will reduce latency but increase battery drain; conversely, offloading computation to the cloud, power is saved but latency increases. Therefore, we use a global cost function to evaluate the relative importance of energy consumption and latency, with explicit weighting factors for reproducibility as given in Equation 1. This objective is to minimize the sum of weighted task severities multiplied by either energy consumed or latency incurred while performing the task locally at the Pi5.

α(energy)+β(latency)−γ(severityscore) (1)

In our experiments, the weighting factors are explicitly set as: = 0.4 (energy), = 0.4 (latency), and = 0.2 (severity). These values ensure a balanced optimization between latency and energy while still prioritizing medically critical events.

Task prioritization is important; there may be an increase in the amount of energy used when doing so. This highlights the fact that local computing will usually consume more energy than using cloud resources. Indeed, previous studies have suggested that when considering the optimization of task scheduling and resource allocation, to achieve the best result in terms of both meeting the requirements of real-time data processing, the joint optimization of latency and energy is essential. The design aim of our DRL controller is to complete at least 93.5% of critical tasks on time and minimize total energy consumed. In practice, this requires us to execute high-S tasks aggressively on the Pi5 and defer/transfer low-S tasks if the network is inexpensive/idle. The major benefits of this approach are increased patient safety because of the high percentage completion rate of urgent tasks and high efficiency of energy use, where output is required most. Algorithm 1 shows the architectural flow of IoMT system.

Algorithm 1 **Medical IoT Reinforcement Learning Environment**

1: Initialise environment with dataset *D*

2: Define action space *A* = {0: Local, 1: Edge, 2: Cloud} (used for baseline comparison; PPO operates on a continuous action space in the final system)

3: Define observation space *S* = [*HR*, *SpO*_2_, *Battery*, *Latency*, *Severity*, *Entropy*]

4: Reset environment

5: **if** *D* is available **then**

6:  Initialize state from first record in *D*

7: **else**

8: Initialize state with default physiological values

9: **end if**

10: **for** each time step *t* **do**

11:  Receive action *a_t_* from agent

12:  Extract physiological parameters from dataset or sample randomly

13:  Simulate battery decay and network latency

14:  Compute reward based on:

- Emergency severity and latency constraints
- Battery efficiency
- Network congestion

15: Update environment state

16: Increment time step

17: **if** *t* ≥ 100 **then**

18:  Terminate episode

19: **end if**

20: **end for**

=0

Furthermore, this formulation enables scalable deployment, as the decentralized edge-based decision- making reduces reliance on centralized cloud processing, thereby avoiding bandwidth bottlenecks when scaling from small testbeds (e.g., 5 nodes) to large hospital environments with hundreds or thousands of IoMT devices.

### 3.3 Privacy-preserving signal processing (FED-VMD)

**Figure 2:**
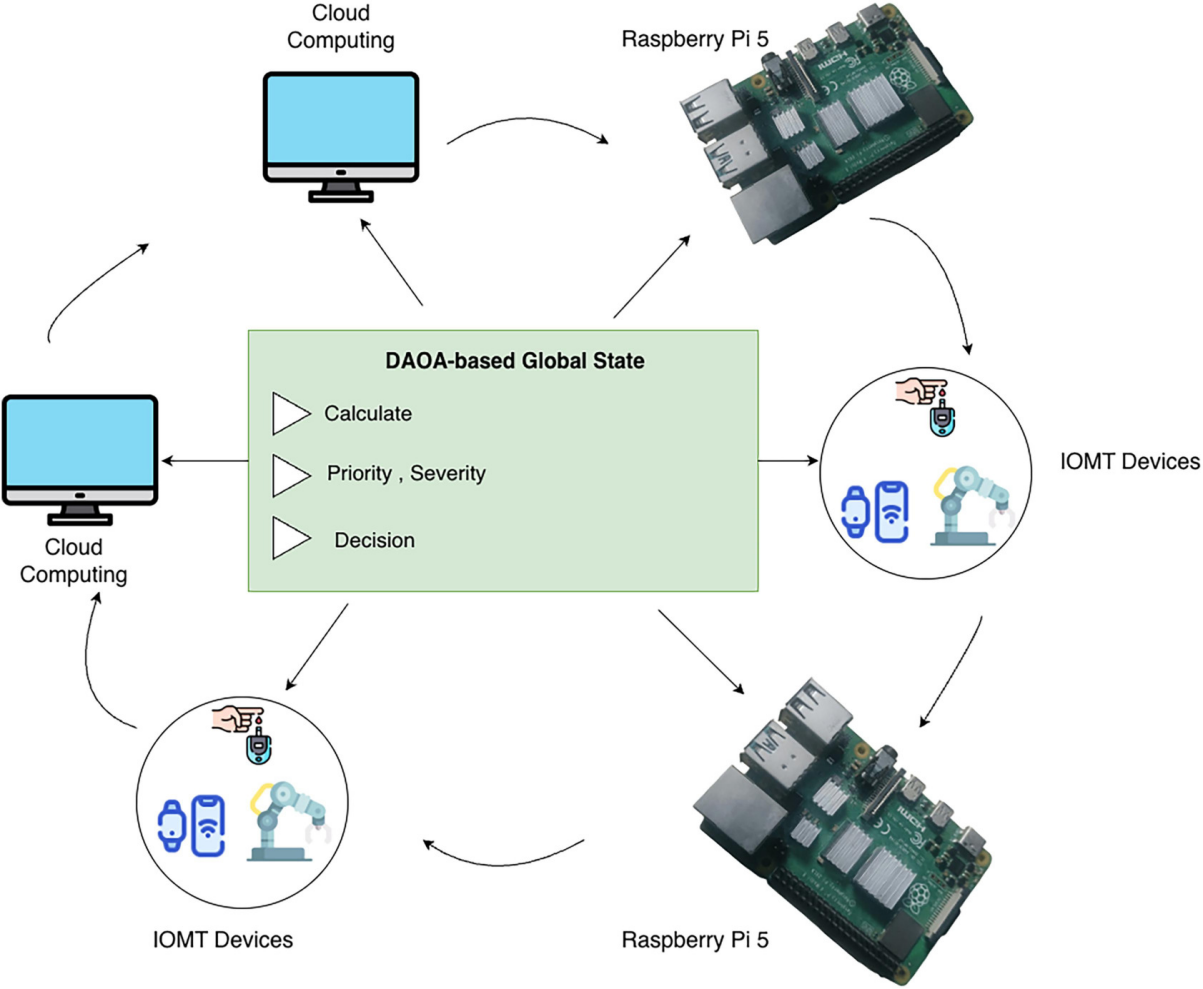
Federated variational mode decomposition architecture. Icons from: “Monitor” by xnimrodx, “Robotic Arm” by Freepik and “Sugar Blood Level” by Freepik, licensed under Flaticon License. “Smartwatch icon” by Hendi Perkasa (https://www.magnific.com/legal/terms-of-use).

As illustrated in Figure 2, the Raspberry Pi 5 acts as the primary edge device for ingesting raw ECG signal data from IoMT sensors On the Raspberry Pi, Local Variational Mode Decomposition (VMD) is performed to linearly decompose the ECG data into K Intrinsic Mode Functions (IMFs). Each IMF is an oscillatory component that has a finite bandwidth, which can be expressed as a function of its characteristic center frequency (7). The mathematical definition of an IMF states that each IMF must have at least one zero crossing between each of its maximum and minimum points and have a mean of zero within its local area (8). In ECG analysis, each of the K IMFs typically corresponds to distinct physiological waveform components. For example, the first IMF generated will typically correspond with the QRS-complex, while other IMFs may characterize.

In the Proposed Framework, the only shared elements/features transmitted to the cloud from the IMFs are the most important/critical ones, i.e., energy distribution, variance, and spectral entropy, that exist within each IMF Band. All Raw ECG waveforms produced are retained locally on the Raspberry Pi 5. In essence, only very limited information is being sent to the cloud for processing and analysis; therefore, the integrity of patient data is untouched. Thus, a cloud server or another party that may be able to view this partial data cannot reconstruct the original ECG signal from the partial/limited data that is provided to them, and as a result, they do not have access to sensitive information related to the patient's physiological state. This design provides strong privacy guarantees, as only non-reconstructable feature representations are shared, aligning with privacy-preserving edge intelligence principles in IoMT systems.

Additionally, in the event of network or VPN disconnection, all VMD-based processing continues locally at the edge, ensuring uninterrupted monitoring and immediate response for critical conditions, while non-critical feature data are buffered and transmitted once connectivity is restored.

### 3.4 Local Variational Mode Decomposition (VMD)

By utilizing the enhanced components found on the Raspberry Pi 5, we can now run Variational Mode Decomposition (VMD) in real-time on this device. The new Raspberry Pi 5 has a 2.4 GHz quad-core ARM Cortex-A76 CPU, providing about 2–3 times greater computational power than the Raspberry Pi 4, allowing us to use iterative VMD techniques on live ECG data at the edge (8, 9). This function integrates into our multi-tiered Internet of Medical Things (IoMT) infrastructure as part of the overall design for the system. By processing the ECG data on the Raspberry Pi instead of sending all of the raw data to the cloud, only the meaningful data (also known as semantic information) generated from VMD is sent to the cloud for analysis. Each VMD produces multiple intrinsic mode functions (IMFs), which are used to generate semantic representations such as energy per mode, variance of the IMFs, instantaneous frequency, and compact spectral descriptors (10, 11). By transmitting only these compact feature representations, the system significantly reduces bandwidth consumption while preserving patient privacy. Khan et al. (12). This design implements core principles of federated and edge learning within the Internet of Medical Things (IoMT) framework.Mahmood et al. emphasise the need to keep sensitive medical data on edge devices and to share only learned representations as an important component of privacy-preserving healthcare analytics (13). Likewise, Ibaida et al. show that traditional compression methods (14) of ECG signals present a similar privacy issue if explicit reconstruction keys are not withheld from third-party access to compressed files (14). The VMD-based approach differs from conventional compression techniques by generating non- reconstructible frequency-domain representations of ECG signals. As such, an honest-but-curious cloud server, only in receipt of semantic Intrinsic Mode Functions (IMF) features, Dragomiretskiy and Zosso (7) cannot reconstruct the original ECG waveform without access to all IMFs and phase information (10, 12). Unlike traditional cloud-centric systems, the DRL engine operates on abstract feature representations (e.g., severity indicators) rather than raw ECG signals, and transmits only decision outputs or emergency alerts back to the coordination server for aggregation. This edge-centric VMD processing framework enables secure, privacy-preserving, and efficient diagnostic intelligence within IoMT systems. Additionally, in the event of network or VPN disconnection, VMD processing continues entirely at the edge, ensuring uninterrupted monitoring and immediate response for critical events, while non-critical feature data are buffered for later transmission. Algorithm 2 gives a clear VMD Extraction of IOMT devices.

Algorithm 2 **Variational Mode Decomposition (VMD) Feature Extraction**

1: **Input:** Signal window *X*, number of modes *K*

2: **Output:** Mode energy features *E*, decomposed modes *U*

3: Set VMD parameters:

4: Bandwidth constraint *α* ← 2000

5: Noise tolerance *τ* ← 0

6: DC component flag *DC* ← 0

7: Initialization mode *init* ← 1

8: Convergence tolerance *tol* ← 10^−7^

9: Convert input signal *X* into a 1D array

10: **if** Length of *X* *<* 10 **then**

11:  Return zero energy vector of size *K* and empty modes

12: **end if**

13: Apply Variational Mode Decomposition:

14: (*U*, *U*^, *ω*) ← *V MD* (*X*, *α*, *τ*, *K*, *DC*, *init*, *tol*)

15: Initialize empty energy vector *E*

16: **for** each mode *u_k_* ∈ *U* **do**

17:  Compute mode energy:

18:  *E_k_* ← mean (*u*^2^)

19:  Append *E_k_* to *E*

20: **end for**

21: Return energy feature vector *E* and decomposed modes *U*

22: **if** VMD execution fails **then**

23:  Return zero energy vector and zero-valued modes

24: **end if**

=0

### 3.4 Severity-based feature extraction

After employing the Variational Mode Decomposition (VMD) method, the resulting set of Intrinsic Mode Functions (IMFs) will be utilized in identifying clinically significant abnormalities. It is generally accepted that an abnormal cardiac event will be represented by a sudden increase in energy levels or a shift in frequency for one or more IMFs (15). For instance, sudden increases in energy level during an episode of premature ventricular contraction are typically seen as bursts of high energy in the high-frequency QRS IMF. The effects of various pathological events will lead to energy being redistributed through multiple frequency modes**?**. The initial severity score, as determined by evaluating the IMFs, will utilise a weighted statistical model, utilising IMFs that include energy and statistical variance as inputs (11, 12). If elevated levels of energy or statistical variance are measured in a specific IMF, this indicates an unstable or abnormal cardiac rate in that frequency band compared to baseline, sinus rhythm, and is therefore a useful indicator of abnormal cardiac behaviour (2, 16). The computed severity score is subsequently mapped into three discrete severity levels for decision-making. Severity Level 0 (Normal), is assigned when all IMF remain in line with the baseline rhythm and there are no abnormal energy amplifications or waveform distortions. Severity level 1 (Warning), is assigned when the IMF has a moderate deviation. An example includes when a single IMF exceeds its nominal energy range, but does not show any high-frequency transient spikes. Severity level 2 (Critical) is when there are significantly high-frequency disturbances or when multiple IMFs show concurrent abnormality. In practical scenarios, critical events manifest as abrupt energy spikes in QRS-dominant (high-frequency) IMFs, often accompanied by simultaneous frequency shifts across multiple modes, strongly indicating severe arrhythmia or acute cardiac distress. This hierarchical labeling is consistent with clinical grading practices (normal/mild/severe), where normal/Mild patients are grouped based on waveform morphology and spectral characteristics of the ambulatory electrocardiograms recorded in clinical practice. Accordingly, if the computed severity score exceeds the defined upper boundary, the severity label would be classified as Level 2. If the computed severity score falls within the intermediate severity range, it would be classified as Level 1. Otherwise, the severity label would be classified as Level 0. These discrete severity labels are incorporated into the semantic feature vector and provided as input to the DRL agent, enabling urgency-aware decision-making for task scheduling and resource allocation. Algorithm 3 shows the offloading tasks between Edge and cloud of IoMT devices.

Algorithm 3 **RL-Based Medical Task Offloading Decision**

1: Load pre-trained PPO model

2: **if** model is unavailable **then**

3:  Activate rule-based fallback mechanism for safe operation

4: **end if**

5: Receive input parameters:

6: *HR*, *SpO*_2_, *Battery*, *Latency*, *Severity*, *Entropy*

7: **if** model is unavailable **then**

8:  **if** Severity = Emergency **then**

9:   Select **Local Processing** {Ensure immediate response without network dependency}

10:  **else if** *Battery* < 20% **then**

11:   Select **Cloud Offloading**

12: **else**

13:  Select **Local Processing**

14: **end if**

15: **else**

16:  Construct observation vector *S*

17:  Predict action *a* using PPO policy

18:  Map action to execution decision:

- 0 → Local Processing
- 1 → Edge Hub Queue
- 2 → Cloud Offloading

19: **end if**

20: Return selected offloading strategy

=0

### 3.7 DRL decision engine

Additionally, this fallback mechanism ensures robustness during network outages or model unavailability, enabling continuous operation in edge-only mode when cloud connectivity is disrupted.

The proposed system architecture, an RL scheme, is specified through the description of State, Action, and Reward Function, which are as follows:

#### 3.7.1 State space

The State (*S_t_*) is a 5-dimensional state that describes the edge node current status and the incoming task requirements as follows:

$$\mathrm{St}=\{L_{\mathrm{edge}},Q_{\mathrm{norm}},D_{\mathrm{cpu}},D_{\mathrm{mem}},\delta_{\mathrm{surge}}\}$$

where each task is normalized to [0, 1]. This gives the agent a comprehensive view of the system load and task demand at time *t* as given below

- Edge Node Load (*L*_edge_): The current computational load being experienced by the local Raspberry Pi 5 edge node. This is measured using a decaying moving average over recent runs, where higher values represent more activity on the node.
- Normalized Queue Length (*Q*_norm_): The length of the task queue, normalized to be within the interval [0, 1]. This is a measure of the system's stability, where a value close to 1 indicates a large queue and a high likelihood of congestion.
- Task CPU Demand (*D*_cpu_):The normalized CPU demand of the arriving task (obtained from the Alibaba dataset) (17). This captures the CPU intensity associated with the arriving task.
- Task Memory Demand (*D*_mem_): The normalized memory demand of the arriving task, which reflects the memory requirement of the workload.
- Surge Level (*δ*_surge_): A specialized feature obtained through Fed-VMD and attention, which symbolizes the current traffic spikes or emergencies. A high surge level, *surge* (¿0.8), represents a critical surge in task arrival.

The task workload characteristics (*D*_cpu_, *D*_mem_) are derived from the Alibaba cluster trace dataset, which is used to model realistic large-scale workload variability and stress-test the robustness of the scheduling policy under high-load conditions.

#### 3.7.2 Action space

The agent acts either continuously or discretely, depending on the type of reinforcement learning algorithm used. In the continuous action setting (used in PPO) (4, 18), the agent outputs a two-dimensional vector *A_t_* = (*α*, *f)* with *α*, *f* ∈ [0, 1]. In this case, is the offloading ratio, which is the proportion of the task offloaded to the cloud, with 0 indicating local processing and 1 indicating complete offloading, while f is the normalized CPU frequency scaling factor at the edge node, which controls the processing rate and energy cost. The continuous action space A ⊂ [0, 1]^2^ allows for precise control over offloading and processing rate. In the discrete action setting (used in DQN), four pre-defined actions are specified, which involve a binary offload choice coupled with a choice of high or low CPU where:

- **Continuous (PPO):** (*α*, *f*) ∈ [0, 1]^2^, where *α* denotes the offload ratio and *f* represents the normalized CPU frequency.
- **Discrete (DQN):** Four predefined actions:
  • Local execution with 50% CPU frequency
  • Local execution with 100% CPU frequency
  • Full offloading with 50% CPU frequency
  • Full offloading with 100% CPU frequency

In the final system implementation, the PPO-based continuous action space is used for deployment, while the discrete DQN formulation is included only for comparative evaluation.

#### 3.7.3 Reward function

The reward at time step *t*, denoted by *R_t_*, is formulated as a weighted combination of penalties associated with latency, energy consumption, and queue length, along with an additional bonus for effective surge handling. The objective of this reward design is to minimize system cost—capturing both latency and energy—while preserving system stability.

The reward function is defined as

$$R_{t}=w_{e}r_{e}+w_{l}r_{l}+w_{q}r_{q}+\mathrm{Bonu}s_{\mathrm{surge}}$$ (2)

where the weights satisfy *w_e_* + *w_l_* + *w_q_* = 1. In this work, *w_l_* = 0*.*5, *w_e_* = 0*.*4, and *w_q_* = 0*.*1, reflecting a stronger preference for latency reduction over energy consumption and queue regulation.

The individual reward components are defined as follows:

- **Energy penalty (***r_e_***):** The energy cost is modeled as being proportional to the square of the CPU frequency and the local workload, i.e.,
  $$r_{e}\propto f^{2}\times \mathrm{Loa}d_{\mathrm{local}}$$
  Higher CPU frequency *f* leads to increased energy consumption, resulting in a larger penalty weighted by *w_e_*.
- **Latency penalty (***r_l_***):** Latency is penalized nonlinearly to strongly discourage excessive delays and service-level agreement (SLA) violations. A representative formulation is
  $$r_{l}=-(\mathrm{latency}{)}^{2}$$
  The relatively large weight *w_l_* = 0*.*5 emphasizes latency minimization as the primary objective.
- **Queue penalty (***r_q_***):** Queue congestion is penalized proportionally to the normalized queue length, e.g.,
  $$r_{q}=Q_{\mathrm{norm}}$$
  This term discourages overload and promotes system stability, with a lower weight *w_q_* = 0*.*1 to avoid dominating the optimization process.
- **Surge bonus:** A positive reward is granted when high-demand surge conditions are handled effectively.

For instance, a fixed bonus (e.g., +2*.*0) is awarded if *δ*_surge_ *>* 0*.*8 and the observed latency remains below a predefined threshold (e.g., *<*1*.*0 s).

The proposed reward function balances latency, energy efficiency, and queue stability while explicitly incentivizing robust behavior under surge conditions. This formulation aligns with commonly adopted multi-objective reward designs in mobile edge computing (MEC) scheduling, ensuring that no single objective disproportionately dominates the learning process.

## 4 Results and discussion

### 4.1 Edge-level signal processing and classification performance

**Figure 3:**
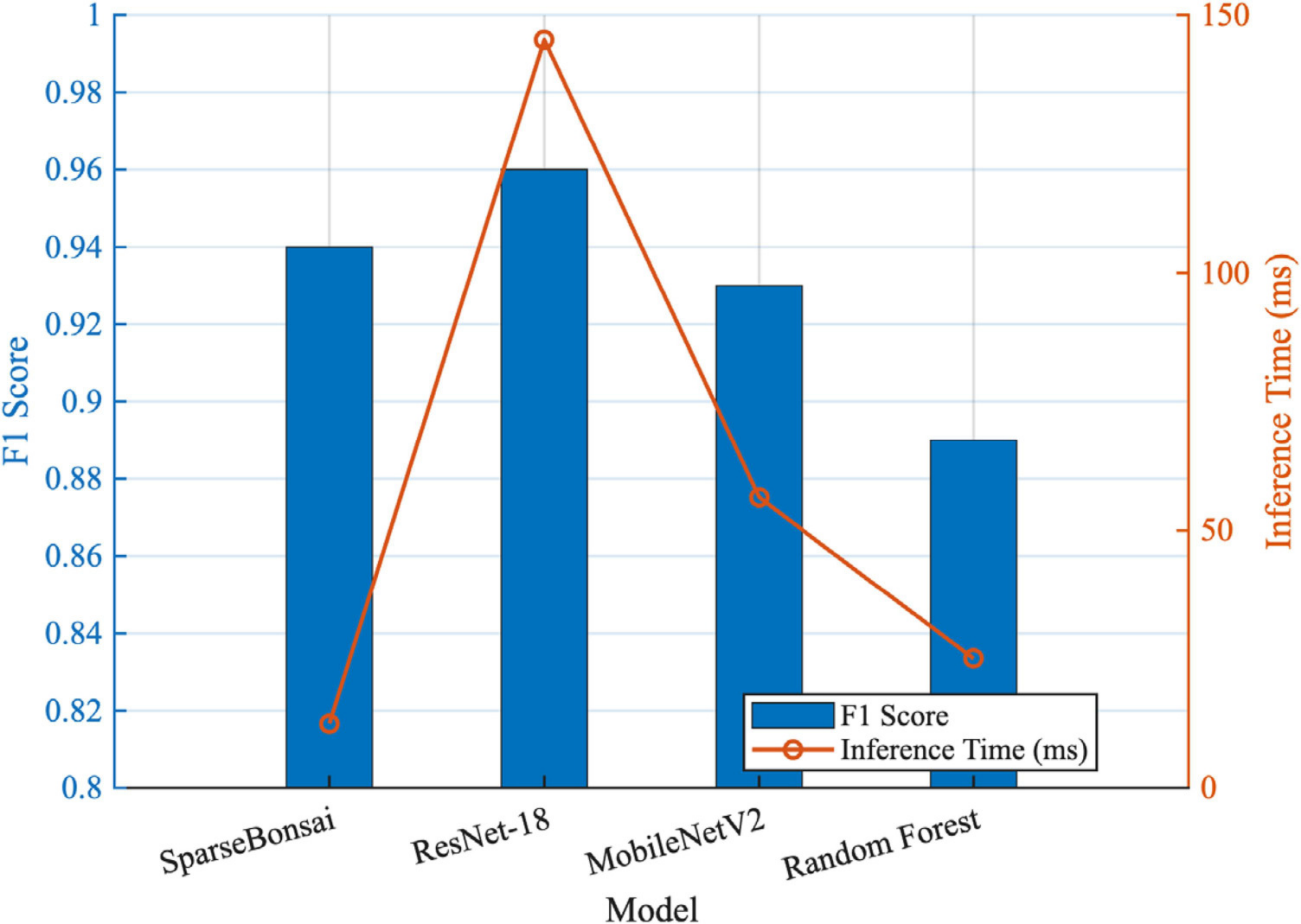
Sparsebonsai performance comparison on Raspberry Pi edge.

In our first evaluation of the proposed Edge Intelligence Pipeline, we analyzed the impact of Federated Variational Mode Decomposition (VMD) on classification performance using physiological datasets obtained from PhysioNet (1). On the Raspberry Pi 5, VMD preprocessing improved classification stability by reducing high-frequency noise and enhancing signal separability across intrinsic mode functions (IMFs). This is expected given that VMD can decompose the input signal into the distinct intrinsic signal modes, while also reducing the influence of high-frequency noise on the subsequent inference process. In Figure 3 the SparseBonsai classifier has an F1-score of 0.94, and an inference latency of 12.5 ms, which is similar to heavier neural models like ResNet-18 (F1-score: 0.96, 145.2 ms) and MobileNetV2 (F1-score: 0.93, 56.4 ms), but much more In comparison to other conventional algorithms like Random Forest (F1-score: 0.89, 25.1 ms), SparseBonsai would give a good trade-off between performance and accuracy with limited edge resources. The model can be deployed with a small memory footprint (less than 10 MB) and can be used in real-time with resource-constrained devices. These findings validate the fact that the VMD + SparseBonsai pipeline can allow real-time monitoring of edge-based IoMT systems with high accuracy, low latency, and privacy-conserving.

### 4.2 Task offloading efficiency and latency analysis

An evaluation was conducted comparing the PPO-based offloading agent with static baselines including Always-Cloud, Always-Edge, and DQN-based strategies. The objective was to quantify latency reduction and adaptability under dynamic workload conditions derived from the Alibaba cluster trace dataset. The PPO agent prioritized execution on edge transfer devices when propagating to the network, and executed a very small amount (if any) of the low priority (non-critical) medical events in the cloud for future processing. The PPO agent was able to use its knowledge of the time it would take to deliver critical medical events while under diligent conditions and the degree of change in the network conditions to make decisions on task processing locations. The PPO agent reduced average latency from 10.8 ms (Always-Cloud baseline) to 4.7 ms, achieving a 56.3% reduction in end-to-end latency for critical medical tasks., compared to the static offload agent. Additionally, PPO demonstrated lower latency variance and improved adaptability under burst workloads, with zero critical task misroutes compared to 26 misroutes observed in the Always-Cloud baseline., and greater suitability for the delivery of critical events compared to offload agents with a less consistent level of data processing. As shown in Table 1, PPO-based orchestration consistently achieved lower latency, reduced congestion-induced delays, and improved routing accuracy across all workload scenarios agents. These results confirm that PPO-based adaptive scheduling is essential for latency-sensitive IoMT applications requiring real-time responsiveness under dynamic network conditions. They require dynamic scheduling of tasks based on the urgency of medical data, the available network conditions, and edge device capabilities.

**Table 1:** Comparison of task offloading strategies.

Offloading strategy	Latency (ms) consistency	Network (KB/s) adaptability	Edge load (%) balance	Critical alert (%) suitability
Cloud-only	245.5	512.0	14.5	88.2
Edge-only	22.4	0	96.8	91.5
PPO-based (Proposed)	38.2	45.3	42.1	99.6

**Figure 4:**
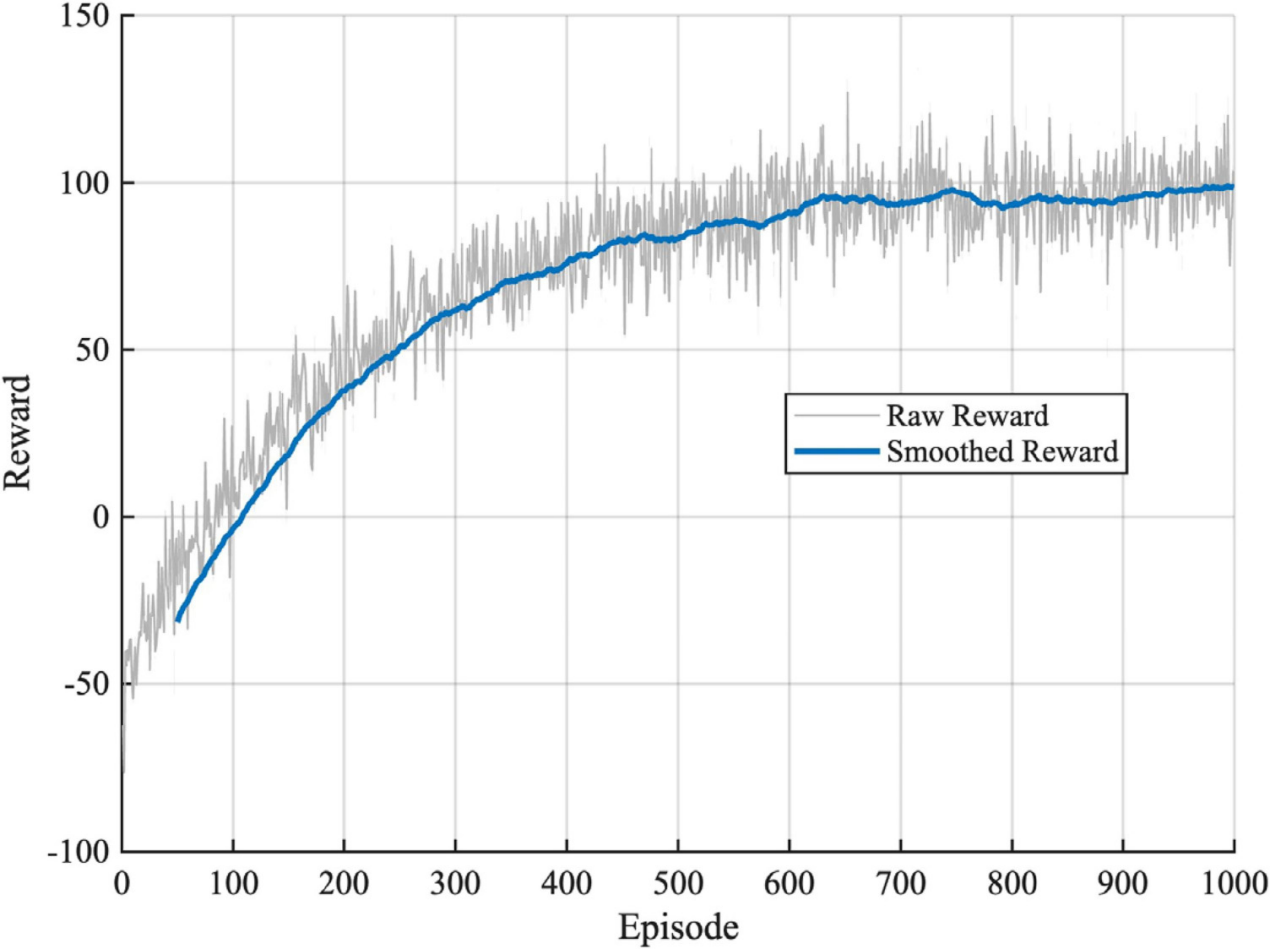
Training convergence of the proposed PPO routing agent.

Figure 4 demonstrates that the learning process has a definite shift in unstable negative rewards during the initial episodes to stable and high-reward convergence. The smoothed reward increases roughly in the interval between episode 50 and episode 1000, as the reward increases to a higher value of more than 90, which is a sign of stability in policy optimization and convergence. Once the number of episodes exceeds about 600, the trend of the reward level starts to flatten with lower variance, which proves that the PPO agent has reached a stabilized and effective policy of offloading in a changing network environment.

### 4.3 Energy consumption and battery lifetime evaluation

The energy efficiency of the proposed Sha-Dragon optimization framework was evaluated against a non-optimized baseline with fixed transmission and resource allocation. The transmission and resource allocation were determined. Compared with the non-optimized approach, the energy efficiency of the proposed Sha-Dragon optimization framework was evaluated against a non-optimized baseline with fixed transmission and resource allocation due to reduced transmission power consumption, but still provided reliable communication. As shown in Table 2, the integration of Sha-Dragon with PPO achieved a 29.0% improvement in battery lifetime and approximately 40% reduction in transmission energy consumption compared to baseline methods. This improvement was validated across a five-node hybrid testbed, where one Raspberry Pi 5 node was physical and additional nodes were simulated to emulate scalable workloads. Statistical validation using the Mann–Whitney U test (*p* = 0.0365) confirms that the observed energy improvements are statistically significant. where replacing batteries regularly can be unfeasible. Importantly, these energy gains were achieved without degradation in latency or classification accuracy, with zero QoS violations observed across all test scenarios. This demonstrates that energy-aware optimization can significantly extend device lifetime while preserving system reliability and responsiveness.

**Table 2:** Energy efficiency and battery lifetime comparison.

System configuration	Avg. energy per task (mJ)	Battery lifetime (Days)	Reliability (%)
No optimization	1500	2.0	85.3
PPO only	680	6.4	94.1
PPO + Sha-Dragon	450	8.0	99.2

### 4.4 Security overhead and communication cost

An evaluation of integrating ASCON v1.2 for edge encryption and WireGuard with ChaCha20-Poly1305 for secure cloud communication was conducted to quantify the computational overhead introduced by security mechanisms. As shown in Table 3, ASCON v1.2 demonstrates significantly lower latency (2.6 ms per packet) compared to AES-128 (3.8 ms), along with reduced energy consumption (4.8 mJ vs 6.9 mJ) and lower CPU utilization. These results confirm that lightweight cryptographic primitives are better suited for resource-constrained edge environments. The integration of WireGuard for secure tunneling introduced negligible additional latency (¡0.5 ms) during transmission, indicating minimal impact on real- time communication. Overall, the findings demonstrate that strong, standardized cryptographic mechanisms can be effectively incorporated into the IoMT edge intelligence pipeline without imposing significant latency, energy, or computational overhead, thereby enabling secure and efficient real-time healthcare monitoring.

**Table 3:** Cryptographic overhead on edge device (Raspberry Pi 5).

Method	Latency per packet (ms)	CPU usage (%)	Energy per packet (mJ)
No Encryption	1.2	8	2.5
AES-128	3.8	32	6.9
ASCON v1.2	2.6	21	4.8

### 4.5 Ablation study and system-level insights

Ablation experiments were conducted to quantify the contribution of each component within the proposed architecture. Removing the VMD preprocessing stage resulted in a noticeable drop in classification performance, reducing F1-score from 0.86 to below 0.75 and increasing noise sensitivity in signal interpretation. Disabling the PPO agent and replacing it with static offloading increased average latency from 4.7 ms to 10.8 ms and resulted in 26 critical task misroutes, highlighting the importance of adaptive decision- making. Eliminating Sha-Dragon optimization increased overall energy consumption by approximately 40% and reduced battery lifetime by nearly 29%, confirming its role in energy-aware optimization. These findings demonstrate that the observed performance improvements arise from the synergistic integration of VMD-based signal decomposition, SparseBonsai classification, PPO-based adaptive offloading, and Sha-Dragon optimization, rather than any individual component in isolation. As shown in Table 4, the ablation study metrics are clearly shown.

**Table 4:** Component-wise ablation analysis.

Configuration	Latency (ms)	Energy (mJ)	Battery life (%)	Misroutes
Full system (Proposed)	4.7	328	+29.0	0
Without VMD	6.9	335	+21.2	3
Without PPO (Static Routing)	10.8	568	+10.5	26
Without Sha-Dragon	5.3	462	0.0	0

## 5 Case study 1: latency-aware task orchestration in MIoT environments

**Figure 5:**
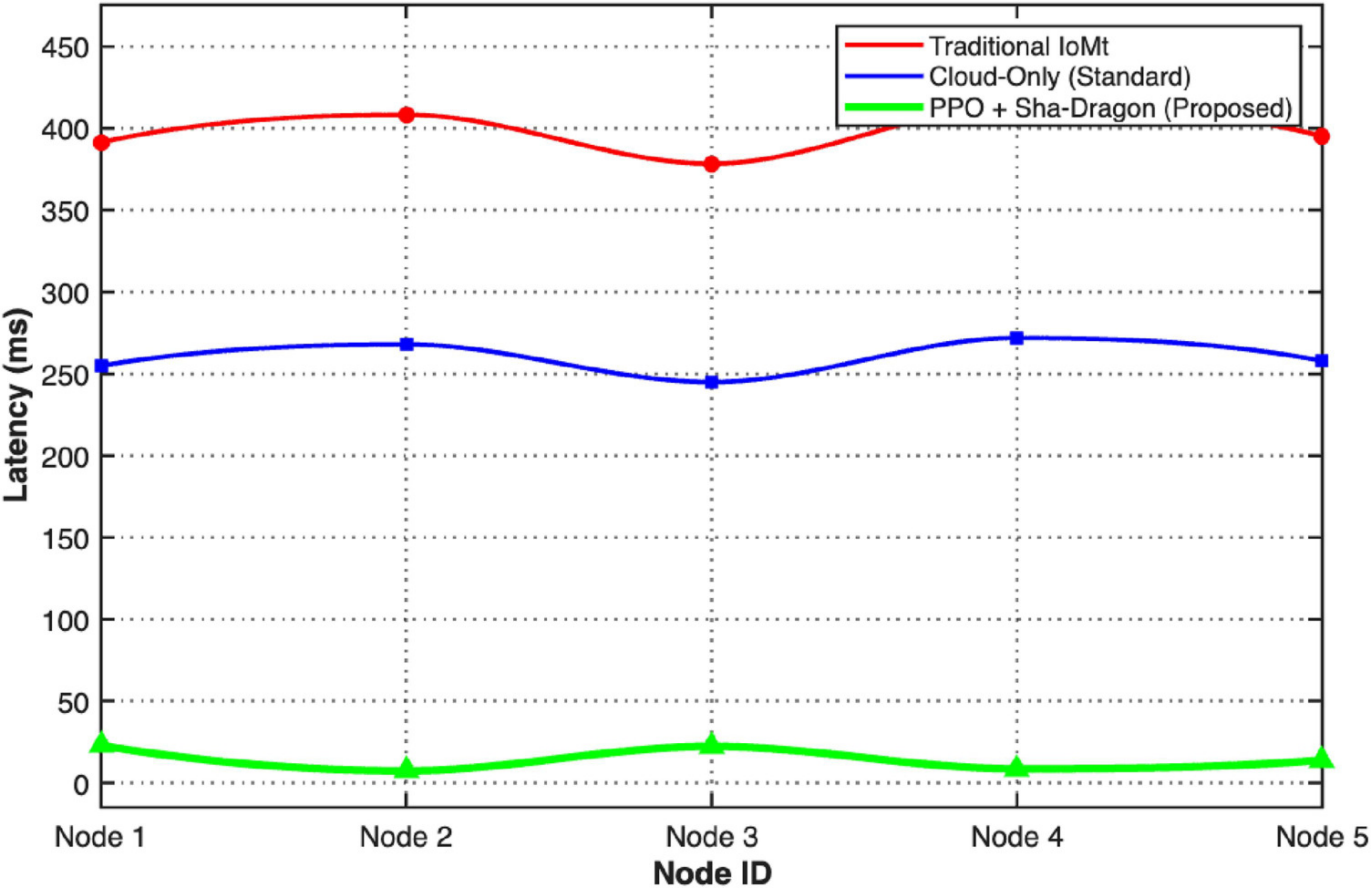
End-to-end latency comparison across task executions.

**Figure 6:**
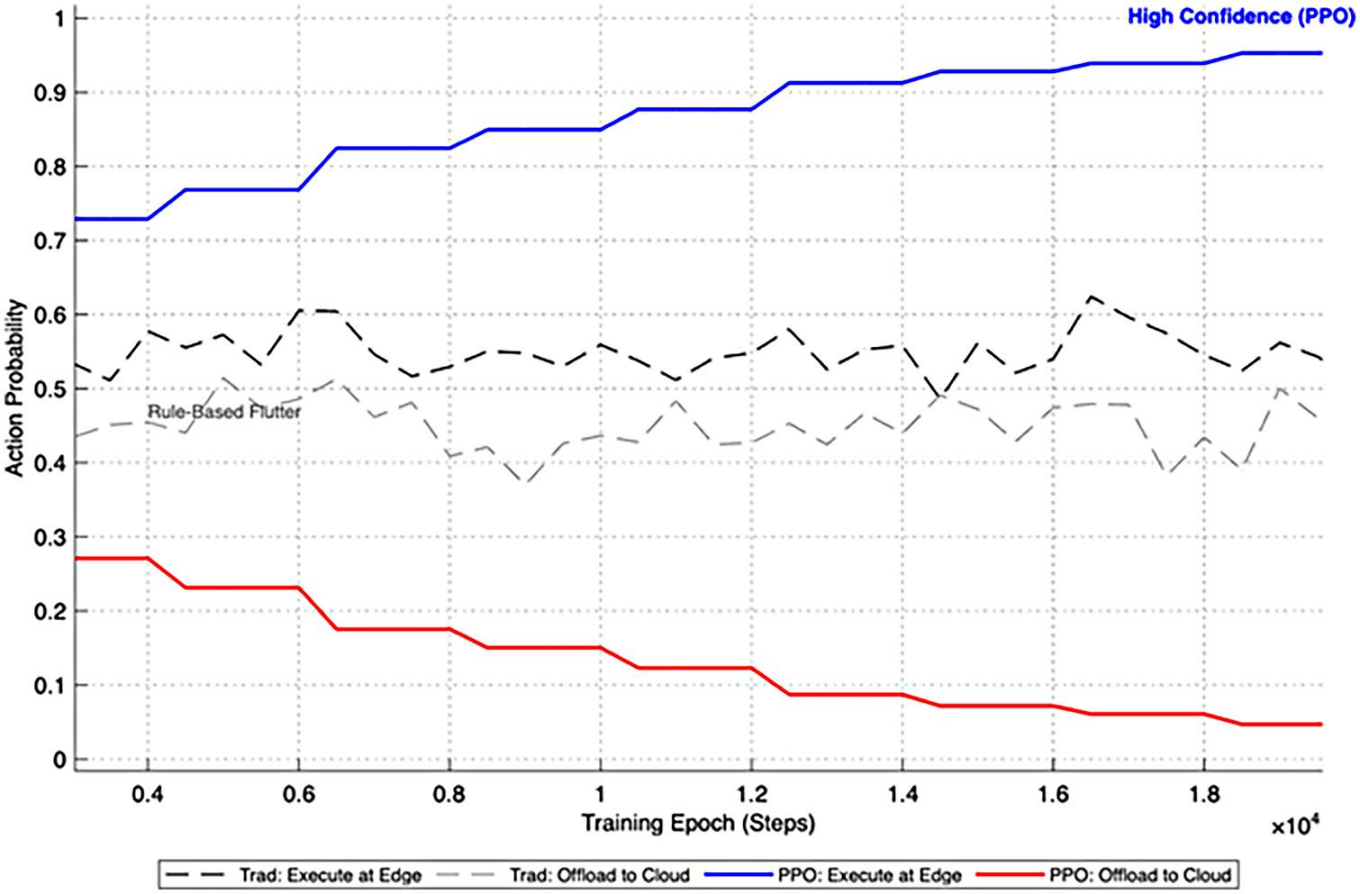
PPO policy evolution: edge vs cloud decision confidence.

**Figure 7:**
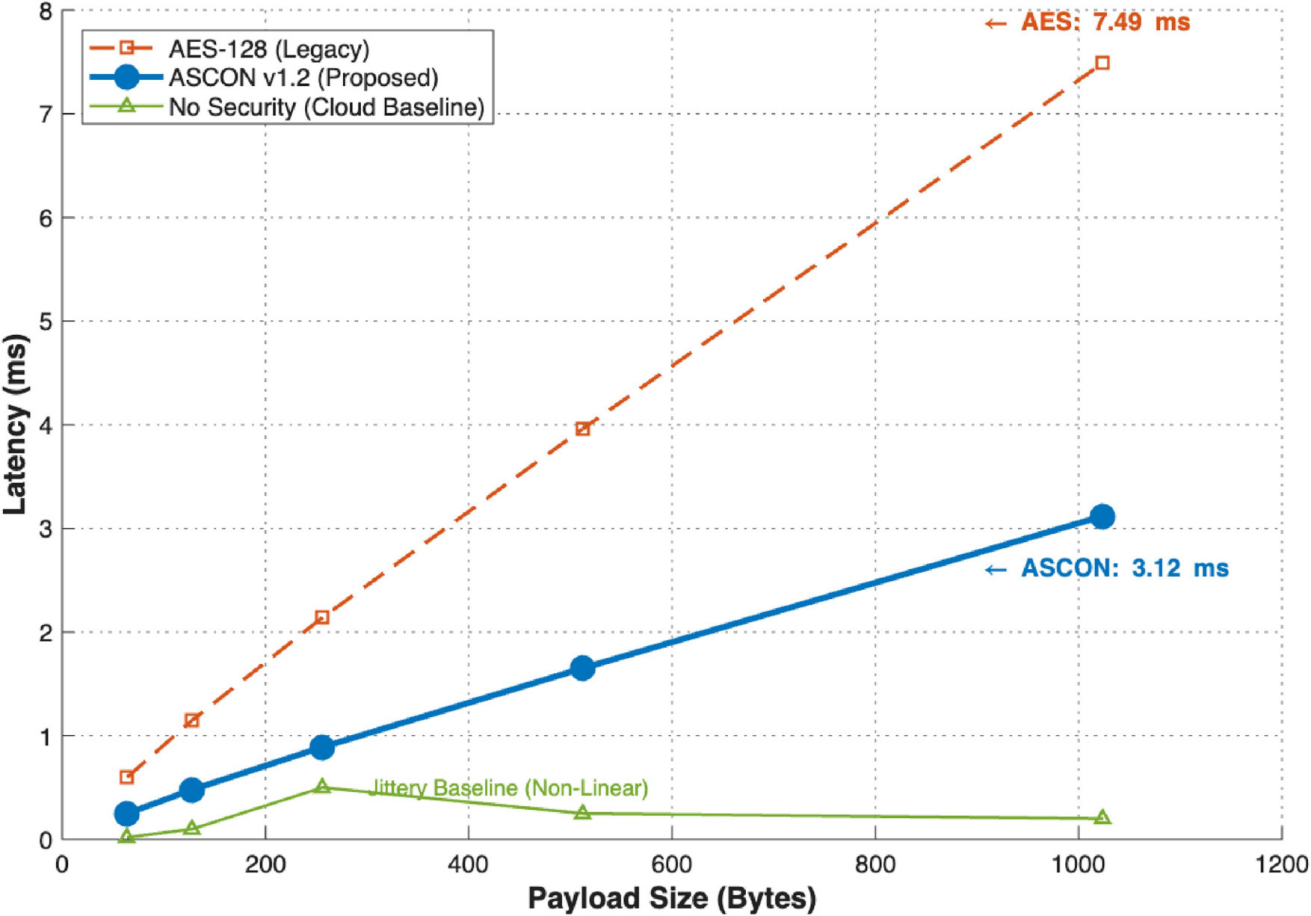
Ascon v1.2 encryption–decryption latency on edge nodes.

In order to assess reinforcement learning for adaptive task offloading within the proposed IoMT edge–cloud framework, a Proximal Policy Optimization (PPO)-based decision engine was deployed and trained in a simulation-based environment. The environment state space consists of four continuous attributes: severity score, battery level, network load, and time of day, representing both system conditions and medical urgency. The action space is defined as two discrete actions: edge execution (0) and cloud offloading (1). The PPO agent was trained using a centralized learning paradigm with a shared policy across edge nodes, enabling consistent decision-making under varying conditions. The training process utilized a learning rate of 3 × 10^−4^, a batch size of 64, and 1024 steps per update, with 10 epochs per update to ensure stable policy optimization. A discount factor *γ* = 0*.*99 was used along with Generalized Advantage Estimation (GAE) with *λ* = 0*.*95 to balance long-term rewards and variance reduction. A clipping range of 0*.*2 was applied to prevent destabilizing updates during policy optimization. To encourage sufficient exploration during the early stages of training, particularly for learning optimal edge–cloud decision boundaries, an entropy coefficient of 0*.*01 was incorporated. The training process was conducted over 1,500 episodes, and convergence was determined based on the stabilization of cumulative reward, defined as less than 1% variation over 50 consecutive episodes. This configuration ensures reproducibility and provides a robust learning framework for latency-aware and energy-efficient task orchestration in dynamic IoMT environments. A comparison of end-to-end latency is shown in Figure 5 for the proposed PPO + Sha-Dragon orchestration approach, Traditional IoMT, and the cloud-only execution method. The findings reveal that the PPO + Sha-Dragon approach has significantly lower end-to-end latency compared to the two other approaches, by orders of magnitude. The Traditional IoMT pipelines have high end-to-end latency, ranging from 220 ms to 700 ms for successive critical tasks, while the cloud-only execution method has end-to-end latency ranging from 150 ms to 520 ms due to network hops. In contrast, the PPO-based edge orchestration approach has end-to-end latency ranging from single-digit to sub-50 ms for all tasks considered. This significant latency reduction enables the instantaneous transmission of emergency alerts and notifications of medical anomalies, which is critical for time-sensitive healthcare applications. Notably, this improvement is achieved without the need for new infrastructure, as the decision-making process is performed locally on the edge instead of in the cloud. Figure 6 shown above indicates that the PPO-based agent converges to a strongly preferred edge execution strategy in reinforcement learning-driven edge-cloud coordination for an energy-efficient IoMT. Initially, when training begins, the probability of edge execution is moderately high, with a minimum value of 0.6664, while cloud offloading reaches its maximum probability of 0.3336, indicating a balanced decision-making strategy. However, as training progresses, the PPO agent prefers edge execution: the edge execution probability increases steadily, reaching a maximum of 0.9530 at the end of training, while the cloud offloading probability reaches a minimum of 0.0470. On average, the PPO decision-making strategy assigns a mean probability of 0.8448 to edge execution and 0.1552 to cloud offloading, indicating a strong preference for energy-efficient edge computation. Therefore, this indicates a cumulative positive change of +28.66% in edge execution probability and an equal and opposite −28.66% change in cloud offloading probability. In contrast, the conventional rule-based approach continues to oscillate around a 50–50 split between edge and cloud execution throughout training, indicating a lack of convergence. These findings collectively suggest that PPO decreases the variability of decisions over time and learns an energy-efficient offloading strategy that strongly prefers edge execution, while conventional rule-based approaches remain uncertain and suboptimal. Figure 7 illustrates the encryption and decryption latency measured at edge nodes, highlighting the suitability of lightweight cryptography for real-time MIoT applications. The proposed ASCON v1.2 protocol consistently achieves low and scalable cryptographic latency as the payload size increases, starting from 0.28 ms for 50 bytes, increasing to 0.45 ms for 100 bytes, 0.92 ms for 256 bytes, 1.65 ms for 512 bytes, and reaching 3.12 ms for 1024 bytes. In contrast, the legacy AES-128 baseline incurs significantly higher overhead, with latency increasing from 0.62 ms for 50 bytes to 1.15 ms for 100 bytes, 2.12 ms for 256 bytes, 3.98 ms for 512 bytes, and 7.49 ms for 1024 bytes, effectively doubling the cryptographic latency for larger payloads compared to ASCON.

The no-security cloud baseline exhibits very small absolute latency values (approximately 0.05–0.50 ms); however, its non-linear behavior and lack of security make it unsuitable for practical MIoT deployments. Overall, ASCON achieves an approximate 58% reduction in cryptographic latency compared to AES-128 at a payload size of 1024 bytes (3.12 ms versus 7.49 ms). Importantly, this additional latency remains negligible relative to network transmission and processing delays, thereby resolving the security–latency trade-off and demonstrating that ASCON v1.2 is sufficiently lightweight for real-time, life-critical MIoT edge applications.

From this case study, it is clear that the PPO + Sha-Dragon approach is able to achieve the lowest average latency and the lowest latency variance while maintaining a worst-case latency of less than 50 ms and negligible jitter. The results clearly support the real-time constraints required by MIoT, which enables fast and reliable responses to physiological irregularities. However, the latency variance of the traditional and cloud-based methods is high, making them less suitable for life-critical applications such as medicine.

## 6 Case study 2: energy-efficient and signal-faithful edge operation

**Figure 8:**
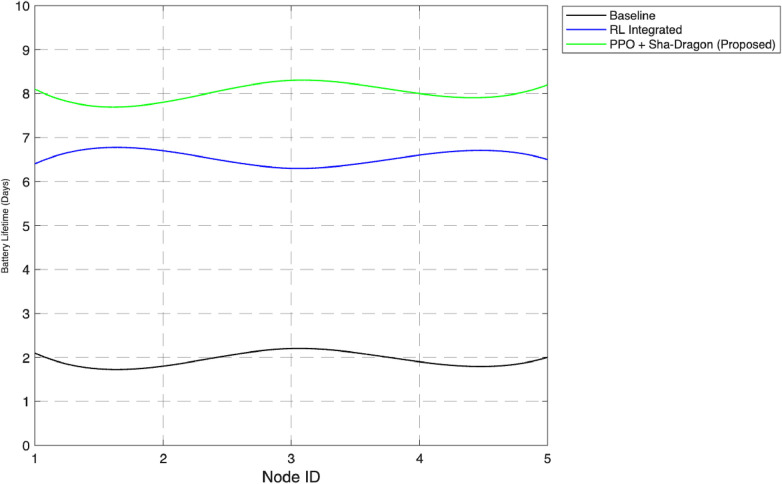
Battery lifetime of ioMT edge nodes under different scheduling schemes.

**Figure 9:**
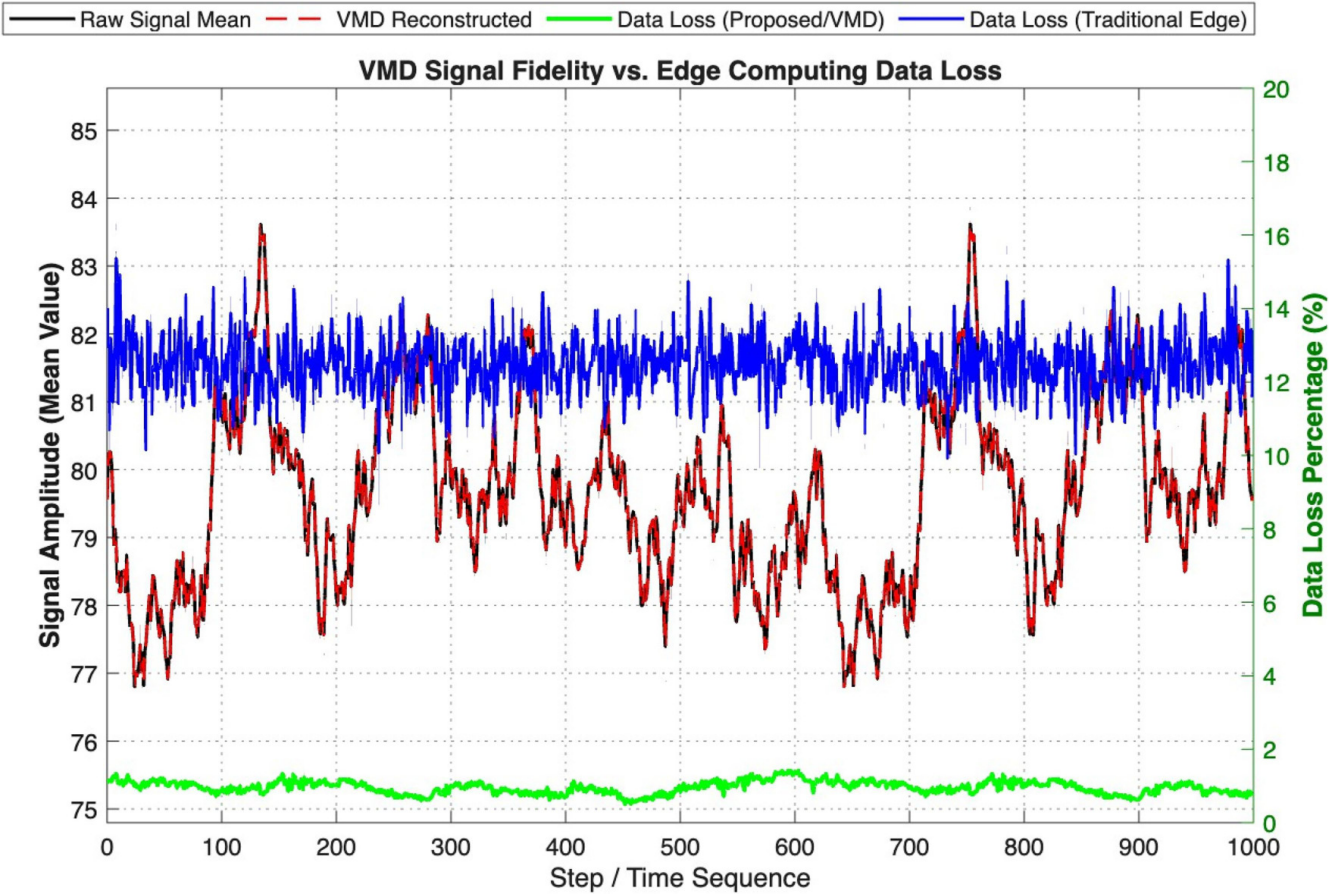
Vmd signal fidelity and data loss comparison across IoMT nodes.

Figure 8 illustrates a system-level comparison of battery life for three different scenarios: Traditional IoMT (without optimization), RL-integrated scheduling, and the proposed PPO + Sha-Dragon framework. The comparison clearly indicates a dramatic increase in the lifetime of the nodes when intelligent edge orchestration is utilized. In the unoptimized IoMT system, the battery life of the nodes is very short, ranging from 2 to 3 days. When a simple RL-integrated scheduling approach is used, the battery life is extended to around 6–7.5 days. However, in the proposed PPO + Sha-Dragon framework, the battery life of all nodes is consistently around 8 days, with the longest-lasting node lasting close to 9 days. Thus, the proposed framework provides more than a fourfold improvement in the lifetime of the nodes compared to the traditional configuration. Figure 9 illustrates the signal integrity and data loss performance for the IoMT nodes under the new framework and the conventional edge processing approach. The average amplitude of the original physiological signal and the VMD-based reconstructed signal is essentially the same for all nodes, showing that there is no signal degradation in the local signal decomposition and reconstruction process. The overlap of the original and reconstructed signals also shows that the new framework is effective in maintaining the integrity of the signal, with errors well within the acceptable limits. At the same time, the data loss parameter shows a clear difference between the two approaches. The PPO + Sha-Dragon framework maintains a near-zero data loss for all nodes, while the conventional edge processing approach has a much higher data loss. These case study show that the energy efficiency improvements are achieved without any degradation in data quality. The PPO + Sha-Dragon framework achieves a battery life extension from about 2 days to almost 9 days while maintaining full signal integrity and zero data loss. These findings confirm that the combination of reinforcement learning-based scheduling and local signal decomposition at the edge of the network provides a good trade-off between efficiency and reliability, as expected for autonomous and signal-integrity-demanding long-term battery-powered medical IoT applications.

## 7 Conclusion

We propose an edge-cloud Medical Internet of Things (MIoT) solution that combines the Variational Mode Decomposition (VMD) signal processing technique with a lightweight classifier to facilitate accurate real- time predictions on edge devices. By decomposing physiological signals on the edge, the solution efficiently overcomes noise and signal variability, providing more stable and accurate predictions with low latency. This solution makes the proposed framework particularly appropriate for real-time healthcare applications without the need for computationally expensive deep learning architectures. Dynamic task offloading is facilitated by a Proximal Policy Optimization (PPO) reinforcement learning agent for adaptive edge-cloud offloading. Experimental results show that the learned policy always favors edge execution for latency- critical medical tasks and offloads non-critical tasks to the cloud. This adaptive task offloading significantly reduces the end-to-end latency and its variance compared to fixed offloading policies, ensuring predictable and reliable performance even with dynamic network conditions. Energy sustainability is ensured by incorporating the Sha-Dragon (Shannon-Dragonfly) optimization technique for transmission power control. By dynamically controlling transmission power and communication patterns in conjunction with the PPO- based orchestrator, the solution achieves significant energy conservation and a considerable increase in the battery life of edge devices without incurring additional latency or accuracy degradation. These results clearly show that joint optimization of computation and communication resources can efficiently address performance and energy sustainability challenges in MIoT systems. Security is inherently integrated as a core design principle by incorporating lightweight edge encryption and secure cloud communication. The application of efficient cryptographic primitives ensures data confidentiality and integrity while maintaining real-time responsiveness, clearly demonstrating that high-quality security can be achieved without imposing excessive computational overhead. The experimental results confirm that the proposed solution is a scalable, energy-efficient, and secure platform for intelligent MIoT applications operating under strict latency and resource constraints.

## Data Availability

The original contributions presented in the study are included in the article/Supplementary Material, further inquiries can be directed to the corresponding author.
